# MicroRNA-492 reverses high glucose-induced insulin resistance in HUVEC cells through targeting resistin

**DOI:** 10.1007/s11010-014-1993-7

**Published:** 2014-02-14

**Authors:** Cai Ying, Liu Sui-xin, Xie Kang-ling, Zhang Wen-liang, Dong Lei, Liu Yuan, Zheng Fan, Zhuo Chen

**Affiliations:** Cardiac Rehabilitation Center, Department of Rehabilitation, Xiangya Hospital, Central South University, Xiang-Ya Road 87, Changsha, 41008 Hunan People’s Republic of China

**Keywords:** miR-492, Insulin resistance, Resistin, Atherosclerosis

## Abstract

The development of atherosclerosis (AS) is a multifactorial process, in which elevated plasma resistin (a key factor leading to insulin resistance) levels play an important role. Emerging evidence indicate that microRNAs (miRNAs) are involved in AS; However, the regulation and function of miRNAs in response to AS remain poorly understood. Our study analyzed the effects of miR-492 on insulin resistance, endothelial activation, and resistin expression in apoE knock-out mice and human umbilical vein endothelial cells after high-glucose treatment and miR-492 mimics transfection. We also investigated the underlying molecular mechanisms. Our results showed that high glucose stress induced a significant decrease in miR-492 expression, with a remarkable upregulation of resistin expression. We then identified resistin as a novel direct target of miR-492 using 3′-UTR luciferase reporter assay. Histopathologic examination demonstrated that upregulation of miR-492 attenuated endothelial cells migration and lipid accumulation induced by high glucose stress. Further investigation demonstrated that the upregulation of p-STAT3, SOCS, and P-selectin activation induced by high glucose stress was attenuated by upregulation of miR-492. Together, our findings indicate that miR-492 contributes to insulin resistance and endothelial dysfunction induced by high glucose, via directly downregulating resistin expression, and involving STAT3 phosphorylation, SOCS, and P-selectin activation.

## Introduction

Cardiovascular disease, such as atherosclerosis, is a critical worldwide health threat, and acts as a leading cause of global morbidity. Emerging evidence suggest that cardiovascular disease is accompanied by changes in serum resistin levels [1]. In addition, there was a positive relationship between plasma resistin and indicators of inflammation and endothelial activation such as leukocyte counts and endothelin-1 (ET-1) levels in blood [2], which atherogenesis was promoted by endothelial dysfunction due to breakdown of the endothelial cell–cell barrier [3]. Resistin was firstly discovered in 2001 [4] and described as a critical factor in development of insulin resistance, which was a major risk factor for atherosclerotic pathogenesis. Recently, it was reported that glucose tolerance and insulin action were impaired by treatment with recombinant resistin in healthy mice, and resistin administration impaired insulin-induced glucose uptake in adipocytes [5]. These findings suggest that resistin is a major inducer of insulin resistance and endothelial damage through the induction of hyper-permeability in human umbilical vein endothelial cells (HUVECs). Furthermore, resistin immunoreactivity is increased in atherosclerotic regions of human aorta and carotid arteries [5]. Suppressor of cytokine signaling (SOCS) carried out a negative feedback loop to decreased signal transduction induced by insulin [6], and signal transducer and activator of transcription (STAT) proteins are involved in SOCS expression in endothelial cells [7]. Although previous studies suggested that resistin caused endothelial dysfunction via activated SOCS3 and JAK/STAT3 pathway, the upstream mechanisms underlying resistin’s action are not fully clear.

MicroRNAs (miRNAs) are endogenous 21–25 nucleotides non-coding RNA molecules that generally bind to the 3′UTR of target genes and repress translation of mRNAs. Recently, miRNAs have been shown to be potential mediators of glucose responses [8]. Moreover, accumulating evidence have demonstrated that miRNAs play crucial roles in endothelial cell function, and numerous miRNAs directly or indirectly control the expression levels of genes involved in glucose stress of HUVECs [9, 10]. It has been proved that prolonged growth of endothelial cells in high glucose-containing medium-induced insulin resistance. Although a recent study suggested that miR-492 played a potent anti-angiogenic role in endothelial cells [11], the regulation and function of miR492 in insulin resistance remain poorly understood.

In this study, we focused on the role of miR-492 in high glucose-induced insulin resistance. We found that the expression of miR-492 was significantly downregulated in apoE-null mice and HUVECs grown in high glucose. Furthermore, we identified resistin as a novel target of miR-492. By suppressing the expression of resistin, miR-492 could inhibit endothelial cells activation partially at least via downregulation of SOCS3/STAT3 signaling pathway.

## Materials and methods

### Animals and cells culture

C57BL/6J wild type and C57BL/6 J apoE knockout (apoE-null) mice were purchased from the Beijing Xie He Medical College (Beijing, China). All animal experiments were complying with the Guide for the Care and Use of Laboratory Animals formulated by the US National Institutes of Health (NIH Publication No. 85–23, Revised 1996) and approved by the Animal Care and Ethics Committee of Central South University. All animals were housed in microisolator cages, and got free access to standard chow and sterile water. Mice (wt, *n* = 5; apoE-null, *n* = 5) were sacrificed at 3 months of age, and blood and aortas were collected. HUVEC CRL-1730 cells and 3T3-L1 adipocytes were obtained from American type culture collection (ATCC). The HUVECs were cultivated in ATCC-formulated of F-12 K Medium supplemented with 10 % FBS, 0.1 mg/ml heparin, 0.04 mg/ml endothelial cell growth supplement. 3T3-L1 adipocytes were cultivated in ATCC-formulated of DMEM with 10 % FBS. All cells cultured in the conditions: 95 % air and 5 % carbon dioxide at 37 °C.

### Antibodies

Rabbit anti-resistin antibody was obtained from Abcam (dilution 1:200). Mouse anti-phospho-Stat3 antibody was purchased from cell signaling technology (dilution 1:100). Rabbit anti-SOCS3 antibody was purchased from cell signaling technology (dilution 1:1,000). Rabbit anti-P-selectin antibody was purchased from Santa Cruz (dilution 1:400). Mouse anti-GAPDH antibody was purchased from Santa Cruz (dilution 1:800).

### Cells treatment

Ectopic expression of miR-492 in cells was achieved by transfection with miR-492 mimics (Genepharma, Shanghai, China) using Lipofectamine2000 (Invitrogen, USA). Insulin resistance HUVEC cells was established by cultivating in F-12 K Medium supplemented 25 mmol/L glucose (5 mmol/L glucose for control group) for 48 h. Cells were plated in 6-well or 96-well plates and transfected for 24 or 48 h. Transfected cells were used for total RNA or protein extraction, and further analysis.

### Haematoxylin-eosin staining

The mice were sacrificed and perfused with saline. The whole aorta was dissected from the body and fixed with 4 % paraformaldehyde overnight at 4 °C, then embedded in paraffin and sectioned (4 μm) for H&E staining. In brief, the sections were deparaffinized and dehydrated in xylene and alcohol, respectively. Then, the slides were stained with Harris’s hematoxylin and eosin stain. Slides were dehydrated, cleared, and mounted. Stained tissues were observed and photographed with an Olympus microscope.

### Oil red O staining

Cells were washed with PBS and fixed with 10 % formaldehyde in PBS for 10 min at room temperature. Cells were incubated with 0.5 % Oil red O solution, and washed with water for 5 min to remove unbound dye. Stained cells were observed and photographed with an Olympus microscope.

### Biochemical analyses

Blood samples were collected from apoE-null and wild type mice after an overnight fast. Fasting plasma glucose was analyzed by glucose oxidase method. Serum insulin concentration was assayed by RIA kit (Beijing North Institute, Beijing, China) following the manufacturer’s protocol. Serum resistin concentration was determined by mouse Resistin ELISA kit (R&D system). The methods of measuring glucose and insulin concentration in cell culture supernatant after treatment were used the same methods as that of in serum.

### Western blot

Tissues or cells were treated with lysis buffer, and 30 μg of total protein was loaded and separated on 10 % SDS-PAGE gel and transferred to nitrocellulose membrane. Immunoblots were incubated with the primary antibodies as the indicated dilution. GAPDH was used as loading control. Signals were detected after incubation with the recommended HRP secondary antibodies using ECL. Images were scanned and analyzed.

### Luciferase reporter assay

The Resistin wild type (Wt) and mutant (Mut) 3′UTR were established and cloned to the firefly luciferase-expressing vector psiCHECK™ (Promega). For the luciferase assay, HUVEC cells were seeded in 48-well plates the day before transfection, and co-transfected with the Resistin Wt or Mut 3′UTR reporter vector, the control vector pRL–TK (Promega), and pre-miR-492 mimics or pre-scramble using Lipofectamine 2000 (Invitrogen). Luciferase activities were measured 48 h after transfection with the Dual-Luciferase Reporter System (Promega).

### Real-time RT–PCR

Total RNA was extracted from indicated cells and freshly frozen aorta dissected from apoE-null or wild type mice with TRIzol reagent (Invitrogen) according to the manufacturer’s instructions. Expressions of resistin mRNA were detected by SYBR green qPCR assay (BioRad, USA). The specific primers are as follow: resistin, F: GCAGGTCTATGCCAGTGTGA, R: TGCCTTGTGGTTCTGTTTGT; β-actin, F: AGGGGCCGGACTCGTCATACT, R: GGCGGCACCACCATGTACCCT. The relative expressions of miR-492 were measured using miRNeasy Mini Kit (Qiagen). The specific primers sets for miR-492 and U6 are as follow: miR-492, F: 5′-TTAGGACCTGCGGGACAAG-3′, R:5′-TTTGGCACTAGCACATT-3′; U6, F: 5′-CTCGCTTCGGCAGCACA-3′, R:5′-AACGCTTCACGAATTTGCGT-3′. All reactions were performed in triplicate. The U6 and β-actin were used as the endogenous controls for miR-492 and Resistin, respectively. The 2^−ΔΔCT^ method was used to process the data.

### MTT assay

Human umbilical vein endothelial cells were seeded at 10,000 cells/well in 96-well plates after glucose treatment and transfection. MTT assay was performed to test cell viability at 0, 12, 24, 48, and 72 h, and the absorbance was measured at 570 nm enzyme immunoassay analyzer.

### Transwell assay

In brief, 5×10^4^ HUVEC cells resuspended in serum-free media were plated into the upper chamber for migration assay after glucose treatment and transfection, and media supplemented with 10 % FBS was filled into the lower chamber. After incubation for 8 h, the cells that had migrated through the membrane to the lower surface were fixed, H&E stained, and counted under the inverted microscope (400×).

### Statistical analysis

T-tests or one-way ANOVA with SPSS 13.0 statistical software were used to analyze statistical data, depending on the experimental conditions. All results are presented as mean ± SEM. Compared with respective controls, *P* < 0.05 were considered statistically significant differences.

## Results

### The expression of miR-492 and resistin in apoE-null mice

**Fig. 1:**
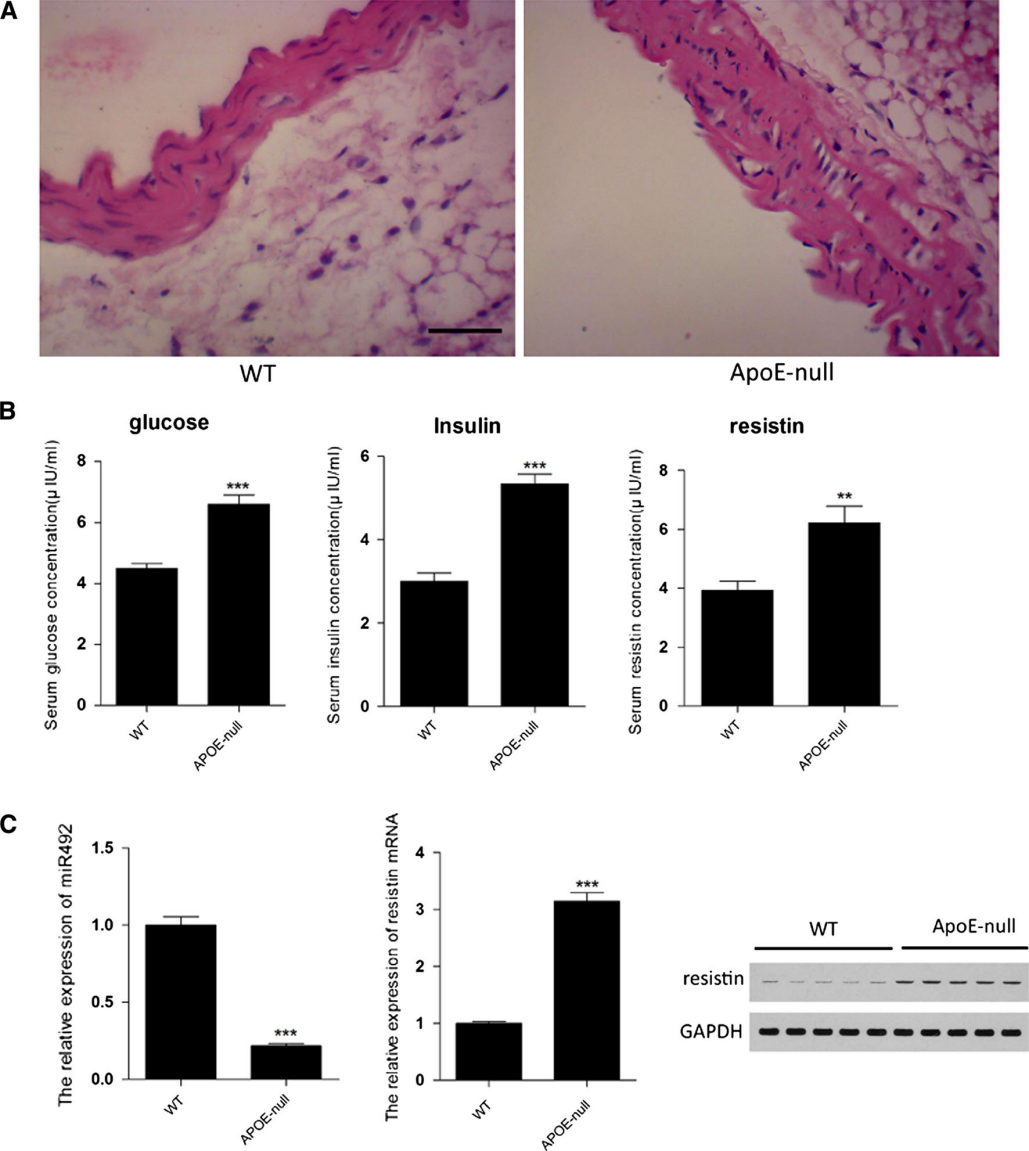
The morphologic differences and the expression of miR-492 and resistin in WT and ApoE knock-out mice. **a** Aorta transverse sections stained with hematoxylin and eosin. **b** Glucose, insulin, and resistin concentration in serum were detected. **c** The levels of miR-492, resistin mRNA in aortic endothelial stripped from WT and ApoE knock-out mice. And western blot analyze the levels of resistin protein. (**P < 0.01, ***P < 0.001 vs. WT group, data shown are mean ± SD.* Bar* 200 μm)

ApoE knock-out mice were widely used in cardiovascular disease research as a classical animal model. The apoE-null mice showed some characters of atherosclerosis and insulin resistance. As the Fig. 1a shown, compare with wild type mice, apoE-null mice showed a thicker vessel wall, vascular smooth muscle were not aligned, muscular layers were under tighten, and foam cells were found between muscular layers. Serum biochemical markers of insulin resistance were detected. We found that glucose and insulin concentration in apoE-null mice serum were increased significantly compare with wild type mice (Fig. 1b), consisting with previous studies [12]. The serum concentration of resistin was also measured, which was upregulated compare to that of wt mice. To determinate the expression of miR-492 and resistin in cardiovascular, aortic endothelial were stripped from WT and apoE-null mice, and the total RNA and protein were extracted. As shown in Fig. 1c, the upregulation expression of resistin mRNA and protein in aortic endothelial tissues was similar to that of in serum, whereas the expression of miR-492 was dramatically downregulated compare to that of wt mice (Fig. 1c). Taken together, we newly found that there was an inverse relationship between miR-492 and resistin in apoE-null mice with pre-atherosclerosis pathological changes and insulin resistance.

### miR-492 directly targets resistin

**Fig. 2:**
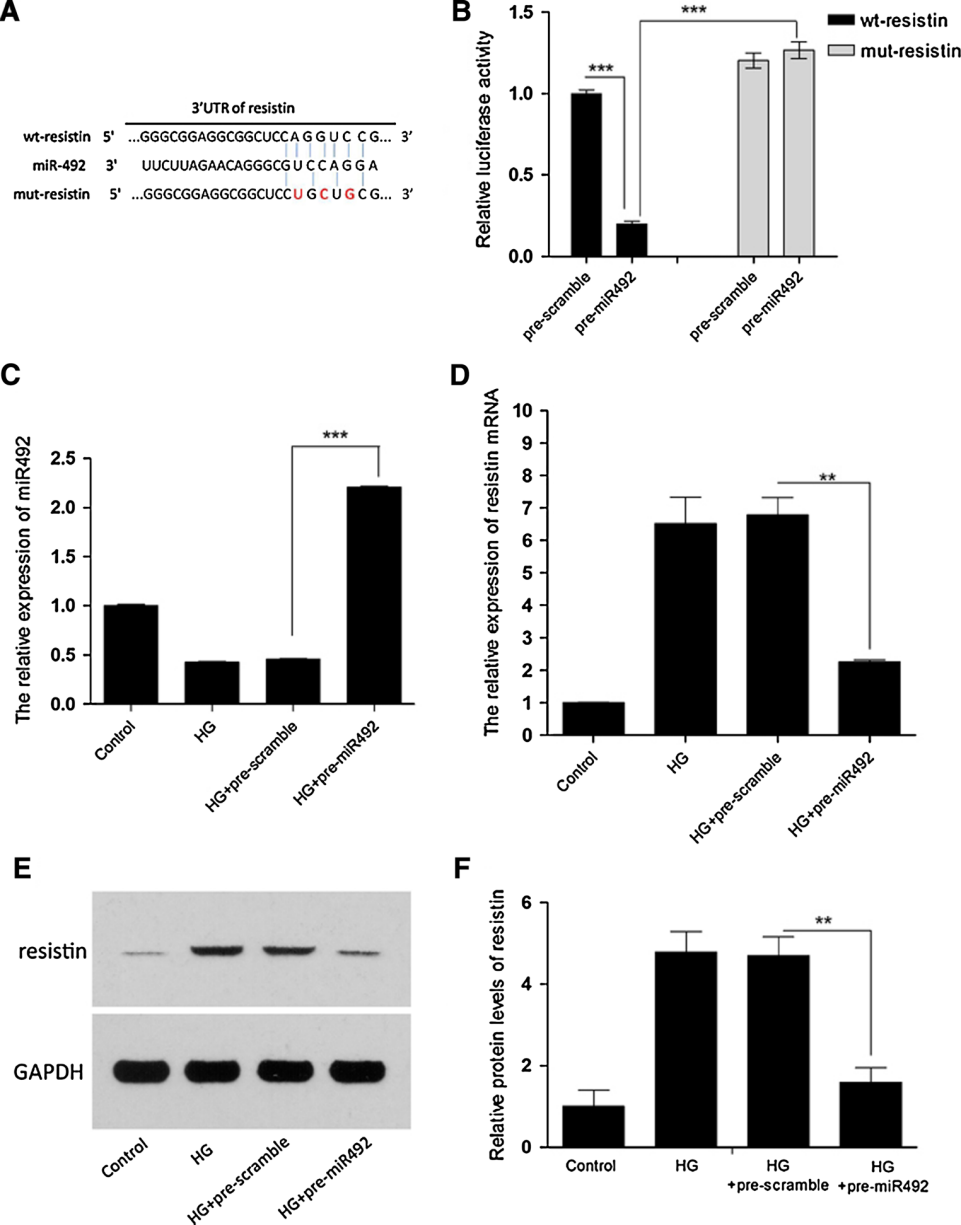
miR-492 directly targets resistin by binding to its 3′UTR. **a** The predicted miR-492 binding site within resistin 3′UTR and its mutated version by site mutagenesis are as shown. **b** The downregulation of luciferase activity by resistin 3′UTR was dependent on miR-492. Mutated resistin 3′UTR abrogated miR-492-induced repression luciferase activity. **c** Reduction of miR-492 by high-glucose treatment was reversed by transfecting pre-miR-492 mimics in HUVEC cells. **d**–**f** Elevated expression of resistin in mRNA and protein levels by high-glucose treatment were inhibited by transfecting pre-miR-492 mimics in HUVEC cells. (*P < 0.05, **P < 0.01, ***P < 0.001 vs. scramble control, data shown are mean ± SD)

Using online miRNA target prediction databases (miTargetFinder and Targetscan), we hypothesized that resistin, an important regulator in insulin resistance, atherosclerosis, and cardiovascular disease, was a target of miR-492 (Fig. 2a). We have examined the resistin and miR-492 expression profile in the aortic endothelial tissues of apoE-null mice. Now we selected HUVEC cells to verify our hypothesis. First, HUVEC cells were cultured in high glucose medium to create the insulin resistance model, and then transfected with miR-492 mimics or scramble (Fig. 2c). Western blotting showed that the enhanced miR-492 in HUVEC cells significantly repressed increase of resistin protein induced by high glucose medium compared to cells transfected with pre-scramble control (Fig. 2e, f). Meanwhile, resistin mRNA expression was also detected by quantitative PCR (Fig. 2d), indicating a potential regulation of resistin by miR-492. To further verify whether the predicted binding site of miR-492-3′UTR of resistin is reasonable for this regulation, we cloned the 3′UTR of resistin downstream to a luciferase reporter gene (wt-resistin); its mutant version (mut-resistin) by the binding site mutagenesis was also constructed. We co-transfected wt-resistin vector and miR-492 mimics or scramble control into HUVEC cells. The relative luciferase activity of miR-492 transfected cells was significantly reduced compared with scramble control (Fig. 2b). In addition, miR-492-mediated repression of luciferase activity was eliminated by the mutant putative binding site (Fig. 2b). These results indicated miR-492 could inhibit resistin expression at transcriptional level.

### Effects of miR-492 on HUVEC cell treated with high glucose

**Fig. 3:**
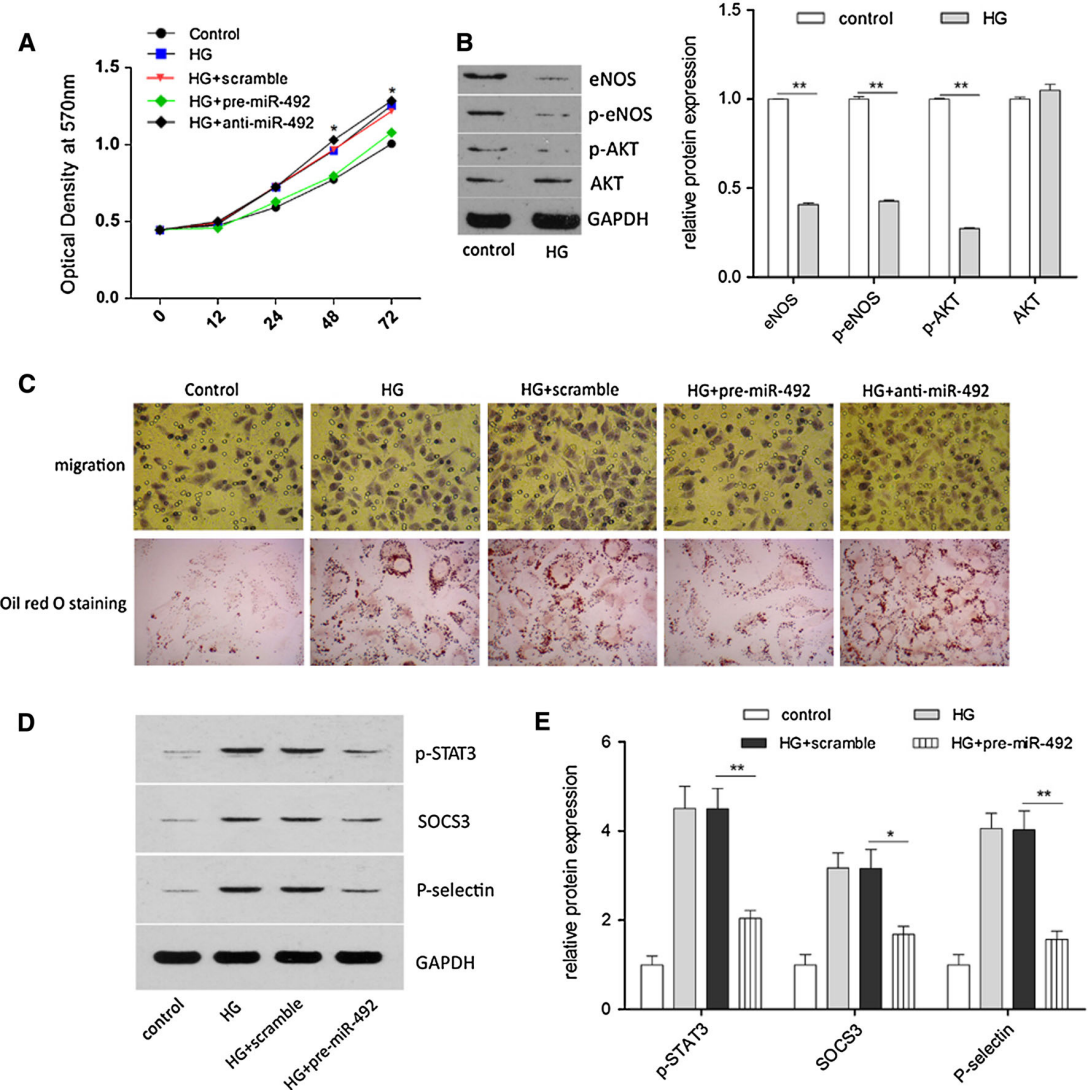
The effects of miR-492 on HUVEC cells treated with high glucose. **a** Ectopic expression of miR-492 by transfecting miR-492 mimics or inhibitors affected proliferation of HUVEC cells, in comparison with scramble controls. **b** Expression of eNOS, p-eNOS, p-AKT, and AKT were detected by western blotting and quantification. GAPDH was used as the loading control. **c** Cell migration of HUVEC cells compared with negative controls. And cells stained with oil red O. **d** Expression of p-STAT3, SOCS3, and P-selectin were detected by western blotting and quantification. GAPDH was used as the loading control. (*P < 0.05, **P < 0.01, ***P < 0.001 vs. control, data shown are mean ± SD.* Bar* 200 μm)

To confirm the insulin resistance model has been established, the expression of eNOS, p-eNOS, and p-AKT were detected. We found that they are significantly decreased by high glucose stress, indicating insulin resistance model has been created. To validate if miR-492 regulates vascular endothelial cell growth, we performed a proliferation assay by transfecting miR-492 mimics or inhibitors into HUVEC cells. It showed that the upregulation of miR-492 induced significant inhibition on cell growth from 24 to 48 h after transfection, whereas the miR-492 inhibitor treatment induced slightly change but not significant (Fig. 3a). Cell motility of transfected cells was also evaluated by transwell assays. As shown in Fig. 3c, compared to the scramble control, miR-492 mimics transfected HUVEC cells exhibited significant impairment of migratory ability, whereas the miR-492 inhibitor treatment had no significant effect on migratory ability of the cells. Oil red O staining was used to evaluate fat accumulation in cells. Compare with scramble transfection, miR-492 transfection resulted a dramatically decrease of fat accumulation in HUVEC cells induced by high glucose culture, whereas more fat accumulation was showed in the cells treated with miR-492 inhibitor (Fig. 3c). To elucidate the probably mechanism on the effect of insulin resistance HUVEC cells induced by miR-492, we characterized STAT3, SOCS3, and P-selectin expression in the insulin resistance HUVEC cells transfected with miR-492 mimics or pre-scramble for 48 h. As representative images shown in Fig. 3d, there were significant inhibition of STAT3, SOCS3, and P-selectin expression transfected with miR-492 mimics in insulin resistance HUVEC cells compare to pre-scramble control.

### Insulin treatment increases the expression of miR-492 and decreases the expression of resistin in 3T3-L1 adipocytes

**Fig. 4:**
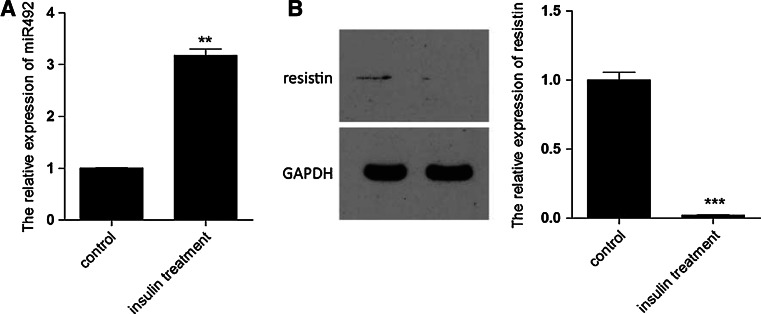
The expression of miR-492 and resistin in 3T3-L1 adipocytes treated with insulin. **a** qPCR detected the expression of miR-492 in 3T3-L1 adipocytes treated with insulin. **b** Expression of resistin was detected by western blotting in 3T3-L1 adipocytes treated with insulin and quantification. GAPDH was used as the loading control. (**P < 0.01, ***P < 0.001 vs. control, data shown are mean ± SD)

In order to further consolidate the regulatory relationship between miR-492 and resistin in insulin resistance, the expression of miR-492 and resistin in 3T3-L1 adipocytes treated with insulin were detected. Upregulation of miR-492 and downregulation of resistin were observed. These findings were similar to the inverse correlation between miR-492 and resistin in aortic endothelial in apoE-null mice (Fig. 4).

## Discussion

ApoE knock-out mice were used as a classical atherosclerosis animal model, which exhibited various syndromes of atherosclerosis such as thicker vessel walls and foam cells formation. Here, we confirmed that apoE-null mice showed some pathology changes of pre-atherosclerosis in aortas endothelial cells, consisting with previous studies [13]. In serum, glucose and insulin concentrations were dramatically upregulated compare to wild type mice, indicating that apoE knock-out resulted in glucose uptake impairment and insulin resistance (IR). Moreover, upregulation of resistin concentration in serum was observed. It was reported that resistin played a pathogenic role in the development of insulin resistance in humans. The increased expression of resistin in mRNA, and protein levels were observed in insulin resistance hepatocytes [14] or peripheral blood mononuclear cells in female patients with diabetes mellitus type 2 compared with healthy women [15, 16]. These findings suggested a positive correlation between elevated serum resistin and insulin resistance.

Insulin resistance is the pathogenic hallmark and strong risk factor for cardiovascular disease, including vascular disease and atherosclerosis. Therefore, further exploration into the molecular biological mechanisms underlying IR will help to the prevention and treatment for atherosclerosis and its complications. Emerging evidence indicated that miRNAs play an important role in atherosclerosis and its related complications [17, 18]. Studies reported that downregulation of miR-145 promotes endothelial lesion formation [19], whereas elevated miR-155 levels are characteristic of atherosclerotic lesions [20]. Little information was available in the literature on the role of miR-492 in cardiovascular disease. The tumor suppressor p53 upregulated the expression of miR-492 in human non-small lung cancer cells [21]. Moreover, downregulation of miR-492 was observed in a micro-array profiling of stage II colon cancers with recurrence of metastases in the liver and/or lungs [22]. These results suggested that there probably was an inverse correlation between the expression of miR-492 and transformation. We discovered that miR-492 was decreased significantly in aortas endothelial tissues obtained from apoE-null mice, while that of resistin was elevated significantly in mRNA and protein levels. These results drove us to explore the relationship between miR-492 and resistin in insulin resistance and even atherosclerosis. We showed that a binding site for miR-492 in resistin 3′UTR was predicted by a bioinformatic algorithm, and the luciferase assays was able to detect a positive miR-492-3′UTR resistin interaction, indicating that resistin was a direct target of miR-492. Furthermore, High glucose stress was used to induce insulin resistance in HUVEC cells [23]. High glucose concentration directly contributes to endothelial dysfunction [24], amplified monocyte adhesion and upregulated P-selectin expression [25]. We found that expression of miR-492 was reduced significantly, whereas the resistin was increased at mRNA and protein levels, similar to the results in aortas endothelial tissues from apoE-null mice. In addition, overexpression of miR-492 by miR-492 mimics transfection was able to abolish the upregulation of resistin induced by high glucose stress in HUVEC cells. Together, we validated that resistin was regulated at transcriptional level by binding its 3′UTR-miR-492.

To further elucidate the effects of miR-492 targeting to resistin in HUVEC cells, we performed a gain-of-function study by enhanced miR-492 expression in high glucose stressed HUVEC cells. The results demonstrated that miR-492 play a suppressive role on cellular proliferation and migration. And what more important was that overexpression of miR-492 effectively reduced the medium glucose and insulin concentrations, indicating an improvement role of miR-492 in insulin resistance. In addition, lipid droplets decreased in number and size after treatment of miR-492 by red oil O staining detection, reversing the upregulation of lipid accumulation by high glucose stress in HUVEC cells. However, the loss-of-function study by knockdown miR-492 expression in high glucose stressed HUVEC cells showed that there were no significant changes compared with the scramble control. Since high glucose stress leads to dramatically downregulation of miR-49, it was probable that anti-miR-492 treatment induced slightly alternations. Moreover, the inverse correlation between miR-492 and resistin in insulin resistance 3T3-L1 adipocytes consolidated their regulatory relationship in HUVEC cells. These findings not only extended the results of previous studies about the effects of resistin on insulin resistance, but also consolidated resistin was a novel target of miR-492.

The role of SOCS3 has been reported as a mediator of the cellular effect of resistin and as a target gene for STAT3 transcription factor in endothelial cells [26]. The studies have shown that in endothelial cells resistin-activated STAT3 pathway and increased SOCS3 protein expression [27], and that SOCS3 was the cellular mediator in the capacity of resistin to antagonize insulin action [28]. It was reported that blocking SOCS3 before stimulation with resistin inhibited the expression of resistin-induced endothelial cell adhesion molecules such as P-selectin [29]. P-selectin played a key role in atherosclerosis and diabetes as a cell adhesion molecule; it was reported that an elevated level in circulating P-selectin was observed in diabetic patients [30, 31] in our recent study, we have verified resistin was targeted by miR-492 and was upregulated at mRNA and protein levels in high glucose stress HUVEC cells. As expectation, our results showed that the enhanced STAT3 phosphorylation, SOCS3, and P-selectin expressions induced by high glucose stress were blocked by miR-492 mimics treatment in HUVEC cells. According to our data and other previous studies, the possible mechanism was that resistin was targeted by miR-492 and upregulates SOCS3 expression by activation of STAT3 pathway, which activated endothelial cell and released adhesion molecules P-selectin.

In conclusion, the present study provides experimental evidence supporting the role of miR-492 in the pathological regulation of insulin resistance and endothelial activation. Our data suggested that resistin, a novel target of miR-492, regulated SOCS3 expression by activation of STAT3 pathway, which activated endothelial cell and released adhesion molecules P-selectin in high glucose condition. These findings indicated miR-492 as a useful molecular target for designing drugs to prevent insulin resistance and/or atherosclerosis. Of note, we now elucidate one of possible mechanisms of atherosclerosis, but the pathophysiology of atherosclerosis involved in many signal transductions, which remain to be determinate in future study.
